# Utilization of Calcium Carbide Residue as Solid Alkali for Preparing Fly Ash-Based Geopolymers: Dependence of Compressive Strength and Microstructure on Calcium Carbide Residue, Water Content and Curing Temperature

**DOI:** 10.3390/ma15030973

**Published:** 2022-01-27

**Authors:** Qiang Wang, Haozhe Guo, Ting Yu, Peng Yuan, Liangliang Deng, Baifa Zhang

**Affiliations:** 1 CAS Key Laboratory of Mineralogy and Metallogeny/Guangdong Provincial Key Laboratory of Mineral Physics and Materials, Guangzhou Institute of Geochemistry, Chinese Academy of Sciences, Guangzhou 510640, China; wangqiang115@gig.ac.cn (Q.W.); guohaozhe@gig.ac.cn (H.G.); yuting@gig.ac.cn (T.Y.); yuanpeng@gig.ac.cn (P.Y.); 2 University of Chinese Academy of Sciences, Beijing 100049, China; 3 Institute of Resource Comprehensive Utilization, Guangdong Academy of Sciences, Guangzhou 510650, China; dengliangliang@gig.ac.cn; 4 School of Civil and Transportation Engineering, Guangdong University of Technology, Guangzhou 510006, China

**Keywords:** calcium carbide residue, geopolymer, C-(A)-S-H gels, fly ash, microstructure

## Abstract

Calcium carbide residue (CCR) is a solid waste resulting from acetylene gas production. In this study, CCR was used as an alkali activator to prepare fly ash (FA)-based geopolymers without any alkali supplementation. We studied the factors (FA/CCR ratio, curing temperature, and water/binder ratio) influencing the mechanical property of FA/CCR-based geopolymers. The compressive strength results showed that, by optimizing these three factors, the FA/CCR mixture has great potential for use as a cementitious material and geopolymer with a dense microstructure having a maximal compressive strength of 17.5 MPa. The geopolymers’ chemical structure, microstructure, and chemical composition were characterized and determined by a combination of techniques. All these results revealed that amorphous C-(A)-S-H (calcium (aluminate) silicate hydrate) gels mainly formed after geopolymerization resulting from the reaction of FA and CCR. In addition, some crystallines, such as ettringite and monosulfate, were also formed. Further, geopolymers prepared with a suitable FA/CCR ratio (1:1 or 1:2) possessed a compact microstructure because of their sufficient reactive SiO_2_ and Al_2_O_3_ and high-enough alkalinity, responsible for higher content of C-(A)-S-H formation and better mechanical property. Too high curing temperature or water content induced the formation of a loosely bound geopolymer matrix that strongly weakens its mechanical property.

## 1. Introduction

Acetylene gas has been widely used in the fields of industry, chemistry, and agriculture. Through the reaction between calcium carbide (CaC_2_) and water (H_2_O) (seen in the following equation), acetylene gas is generated [1].

(1) $${\mathrm{CaC}}_{2}+2H_{2}O\to \mathrm{Ca}{\left(\mathrm{OH}\right)}_{2\;}+C_{2}H_{2}$$

Yet, substantial industrial waste, called calcium carbide residue (CCR), is also generated during the production of acetylene gas, often in large amounts because of the high demand for acetylene gas; it is estimated that China alone produces 28 million tons of CCR annually [2]. The CCR is mainly composed of calcium hydroxide, which has high alkalinity [3,4,5], so the accumulation of considerable high-alkaline CCR has a detrimental role in the environment and ecology [6], via its water alkalization as well as biological impacts [7]. In addition, CCR often contains heavy metals, whose migration during rains can lead to heavy metal pollution in both the soil and water. However, CCR is generally processed by burial, stacking it in open air, or even pouring it in the sea because of high transportation costs and few proven ways of utilizing it for another purpose, all of which pose serious environmental problems [2,6]. CCR has been studied for waste gas and wastewater treatment, adsorption of ions [8], soil treatment [9], and CO_2_ capture [10]. Some studies [11,12,13] have focused on the comprehensive utilization of Ca-containing solid waste, which efficiently solidified the heavy metal and produced an inert and eco-material. Based on these studies, combining the properties of CCR, it is imperative finding a feasible way of reusing CCR to derive environmental, social, and economic benefits from this widespread by-product [14].

In this respect, CCR has the potential of serving as substitute for raw materials of ordinary Portland cement (OPC) [15,16]. Being one of the most widely used construction materials, OPC nonetheless releases large amounts of carbon dioxide (CO_2_), consumes plenty of energy, and produces large quantities of dust during its production. According to several studies, to produce 1 t OPC consumes approximately 3300 MJ of energy, emits approximately 1 t CO_2_, and generates approximately 10,000 m^3^ dust [17,18]. Even a partial replacement of OPC raw materials with CCR should reduce the carbon footprint, which would alleviate the environmental burden of both CCR and OPC, to a certain extent. However, as the replacement level of CCR is increased, the mechanical strength of OPC is decreased [16]. Therefore, to ensure a robust strength level, the degree of substitution of CCR for OPC’s raw materials must be kept to a low level. Accordingly, environmental pollution arising from OPC production remains serious due to the continued low utilization rate of CCR.

Alkali-activation geopolymer is a new type of cementitious polymeric material consisting of [AlO_4_] tetrahedrons and [SiO_4_] tetrahedrons linked by bridging O atoms; it is considered a strong competitor to OPC due to its low environmental footprint and excellent characteristics, namely its excellent mechanical properties, strong interface bonding strength, and high durability [19,20,21,22]. Generally, a geopolymer can be synthesized by mixing of an aluminosilicate (e.g., halloysite and fly ash) with an alkaline solution (e.g., NaOH and Na_2_SiO_3_ solution), followed by a curing process [23,24,25]. In particular, fly ash (FA), a by-product generated from the combustion of pulverized coal in coal-fired electric power plants, is another large-scale solid waste that harbors harmful trace elements and thus requires proper disposal [26,27]. Nevertheless, FA consists mainly of reactive SiO_2_ and Al_2_O_3_, these proven to possess high pozzolanic activity and capable of acting as an additive to OPC [28,29]. FA is known as an important aluminosilicate precursor for geopolymer preparation: when attacked by alkali, FA will release free [SiO_4_] and [AlO_4_]^−^, following by polymerization to form the -Si-O-Al-O- network [30]. For this reason, FA-based geopolymers have been widely studied and applied in civil construction projects, like airport runways and buildings [31].

Calcium (Ca) can accelerate the geopolymerization process and also compact the microstructure, thereby improving the mechanical properties of a geopolymer [32,33]. Not surprisingly, some studies have recently focused on adding CCR to replace some FA in the process of geopolymer preparation given the existence of Ca in CCR. For example, Tanakorn et al. [34] prepared a sustainable repair material by harnessing the alkali-activation of high-calcium FA mixed with CCR. They found that the setting time quickened with more CCR used and a higher Na_2_SiO_3_/NaOH ratio, while the compressive strength initially rose but then diminished as the CCR replacement level and Na_2_SiO_3_/NaOH ratio increased. Although FA and CCR have been proven to function as suitable precursors for generating an alkali-activation geopolymer, the use of a high alkaline solution is rather costly financially and the replacement level of CCR is still relatively low.

Considering that CCR is a highly alkaline residue that mainly consists of CaO, accompanied by some SiO_2_ and Al_2_O_3_, it appears that CCR could be used as solid alkali to activate FA [35]. Both FA and CCR can mix well when dry, and “just adding water” enables the preparation of homogeneous geopolymeric pastes after stirring [1,36]. The chemical reaction between FA and CCR is similar to the pozzolanic reaction and one of its final products is calcium silicate hydrate (C-S-H) [1,37]. Therefore, it would seem feasible to prepare a FA/CCR-based geopolymer concrete without the addition of extra alkaline solution. Nattapong et al. [1] studied the effects of binder contents and water/binder ratios on the compressive strength of FA/CCR-based geopolymer concrete, in which ratio of FA/CCR used was 70:30. The highest compressive strength was attained at 24.3 MPa when the binder content was 450 kg/m^3^ with a water/binder ratio of 0.35. Work by Dueramae et al. [36,38] investigated the compressive strength and autogenous and drying shrinkages of FA/CCR-based geopolymer mortars. Increasing the curing temperature favored the development of compressive strength and reduced drying shrinkage, while the addition of NaOH was able to improve the mechanical properties but it increased the autogenous and drying shrinkages. Evidently, most of the studies to date have focused on the mechanical properties of FA/CCR-based geopolymer mortar or concrete [2,39,40,41,42], including it compressive strength, flexural fatigue strength, water permeability, and bonding strength, to name a few. There is a dearth of research on the microanalysis of the FA/CCR-based geopolymer itself. Arguably, more in-depth studies of FA/CCR-based geopolymers are required, vis-à-vis the chemical composition, microstructure, and mechanical properties of geopolymeric gel, which is of timely significance for the better utilization of CCR and development of “green” geopolymeric materials.

In this work, we prepared geopolymers using FA and CCR as their raw materials. The effects of curing temperatures, FA/CCR ratios, and water/binder ratio on the chemical composition, microstructure, and mechanical property of this suite of geopolymers were investigated. Then different analytical technologies—X-ray diffraction (XRD), thermogravimetric analysis (TGA), Fourier-transform infrared (FTIR) spectroscopy, and scanning electron microscopy (SEM) —were used to study the microstructures and properties of the FA/CCR-based geopolymers. This work can provides better insight into geopolymerization of CCR and FA under different preparation conditions.

## 2. Materials and Methods

### 2.1. Characterization Methods

To determine the chemical composition of raw materials, their XRF was recorded on a wavelength-dispersive sequential scanning spectrometer (Shimadzu XRF-1800, Kyoto, Japan). In addition, the particle size distribution of raw materials was determined, this using a JL-1177 laser particle size analyzer (Chongqing, Sichuan province, China). Compressive strengths for different ageing days (1 d, 7 d, 14 d, and 28 d) of FA/CCR-based geopolymers, as prepared by different conditions (Table 1), were determined by unconfined compression tests run on a YAW-300D Compression Resistance Tester (Lixian, Dongguan, Guangdong province, China).

The XRD patterns were collected on a Bruker D8 Advance diffractometer (Mannheim, Germany) to analyze the mineralogical phase of raw materials and the geopolymers. To identify changes to their structure after geopolymerization, FTIR spectral data were collected on a Bruker Vertex 70 spectrometer (Karlsruhe, Germany). The TGA and DTG curves were recorded with a Netzsch STA 409PC instrument (Selb, Germany) to test the thermal stability and composition of CCR and geopolymers. Finally, to observe the morphology and microstructure of raw materials and geopolymers, SEM images and EDX spectroscopy were recorded using an SU8010 field-emission scanning electron microscope (FESEM, Hitachi, Japan).

### 2.2. Materials

Fly ash (FA) was provided by Foshan Hengyi power plant while the calcium carbide residue (CCR) came from the Foshan Jinrongtao Gas Product Co., Ltd. in Foshan, Guangdong Province, China. The chemical composition of FA and CCR, as measured by X-ray fluorescence (XRF), is presented in Table 1.

**Figure 1:**
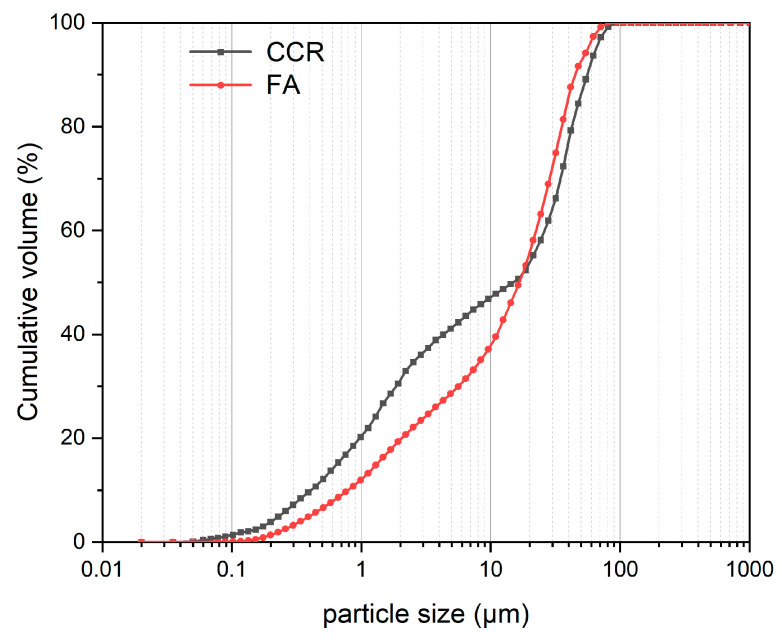
Particle size distributions of FA (fly ash) and CCR (calcium carbide residue).

Clearly, FA contained high amounts of SiO_2_ and Al_2_O_3_, these amounting to 75.3% of its total content. In addition, the CaO content of 10.8 wt% indicated this sample was a Class C FA. The CCR was primarily composed of CaO, accounting for 94.8% of the total content. Figure 1 depicts the particle size distributions of CCR and FA; their respective median particle size was 27.13 μm and 21.89 μm.

### 2.3. Preparation of Geopolymers

To prepare the geopolymers, CCR and FA were used as solid raw materials with ultra-pure water as the liquid raw material. Before using them in this study, these solid raw materials were both dried at 80 °C for 48 h. The CCR was pulverized in a mortar by hand and then passed through 200-mesh sieve.

First, different ratios of CCR and FA were mixed together by a cement mixer for 5 min. Then, different contents of ultra-pure water were added into the cement mixer for mixing with the solid raw materials, to form a homogeneous paste. Each mixed paste was cast into a silica mold (20 × 20 × 20 mm^3^), and then all the filled-in silica molds were covered with a thin polyethylene film to prevent the evaporation of water. Next, the specimens were cured in an oven at 80 °C, for 1 h, followed by their de-molding and storing in sealing bags. These specimens were further cured at different temperatures until their day of testing. The experimental conditions are detailed in Table 2. The products obtained were denoted as FAXCCRY-L-T, where X:Y is the weight ratio of FA:CCR, L is the water/binder ratio, and T denotes the curing temperature. For example, FA1CCR2-0.4-50 °C is the geopolymer specimen produced with a FA/CCR ratio of 1:2, a curing temperature of 50 °C, and a water/binder ratio of 0.4.

## 3. Results and Discussion

### 3.1. Compressive Strength of FA/CCR-Based Geopolymers

#### 3.1.1. Effect of the FA/CCR Ratio

**Figure 2:**
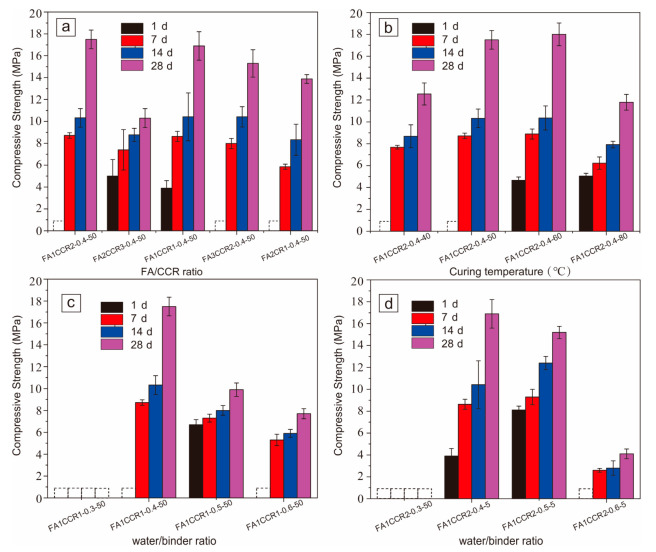
Effects of different factors on the compressive strengths of FA/CCR-based geopolymers. The (**a**) FA/CCR ratio, (**b**) curing temperature, and (**c**,**d**) water/binder ratio.

The mechanical properties of FA/CCR-based geopolymers are highly dependent on the FA/CCR ratio used in their preparation. As Figure 2a shows, only the FA2CCR3-0.4-50 °C and FA1CCR1-0.4-50 °C featured 1-d compressive strength; either excessive FA or CCR led to generally this low level of early compressive strength. From previous studies [1,38], we know that FA provides reactive SiO_2_ and Al_2_O_3_, whereas CCR provides an alkaline environment during geopolymerization. In our study, when the FA/CCR ratio was lower than 1:2, there was insufficient [SiO_4_] and [AlO_4_] oligomer at the early stage of geopolymerization. When the FA/CCR ratio was higher than 3:2, the alkalinity became weaker, leading to little dissolution of FA. The dissolution of aluminosilicate is the first important step of geopolymerization, so this low alkalinity also resulted in insufficient [SiO_4_] and [AlO_4_] oligomers. Therefore, these specimens could not gain compressive strength at 1 d.

As the ageing time increased, the compressive strength of all specimens increased. This result demonstrated that the longer geopolymerization went on, more geopolymeric gels formed, thus improving the compressive strength. The geopolymers FA1CCR2-0.4-50 °C and FA1CCR1-0.4-50 °C had the highest 28 d compressive strength, being approximately 17.5 MPa. The 28 d compressive strength decreased slightly as the FA/CCR ratio increased from 1:1 to 2:1. However, considering the need to better dispose of CCR, the FA/CCR ratio of 1:2 was selected for closer examination.

#### 3.1.2. Effect of Curing Temperature

In Figure 2b is the compressive strength of the FA/CCR-based geopolymers cured at differing temperatures. We can see that the 1-d compressive strength increased with an increase in the curing temperature used. This is because increasing the temperature accelerated the dissolution, polycondensation, and consolidation of FA/CCR in the early stage of geopolymerization [19,43]. However, the 28-d compressive strength increased at first and then decreased sharply as the curing temperature rose from 40 °C to 80 °C; this result indicated a high curing temperature does not favor the development of compressive strength for FA/CCR-based geopolymers. This could be attributed to the rapid setting and hardening of the geopolymer when cured at a too-high temperature, without sufficient dissolution, leading to less geopolymerization. Furthermore, higher curing temperatures produced the geopolymer with a more porous structure because of a more rapid evaporation of water [19,44]. Considering the energy input, the curing temperature of 50 °C was selected for closer study.

#### 3.1.3. Effect of the Water/Binder Ratio

The mechanical properties of FA/CCR-based geopolymer were also influenced by the water/binder ratio used. Figure 2c,d show the compressive strength of FA/CCR-based geopolymers prepared with different water/binder ratios for FA1CCR2-L-50 °C and FA1CCR1-L-50 °C, respectively.

When the water/binder ratio was lower than 0.4, the water content was too low for the geopolymeric paste to gain adequate workability. Therefore, these specimens had no compressive strength. Increasing the liquid/solid ratio increased the workability of geopolymeric paste and accelerated the dissolution of aluminosilicates, which increased the early compressive strength of as-obtained geopolymers. However, using a high liquid/solid ratio may also lead to excess water, which could reduce the alkalinity of the reaction system, decrease the polycondensation of aluminosilicates, and generate more pores during curing, thus resulting in reductions to late compressive strength [1,45]. Accordingly, when the water/binder ratio was augmented, from 0.3 to 0.6, we found the 28-d compressive strength increased from 0 to 17.5 MPa but then it decreased to 8.5 MPa for FA1CCR2-L-50 °C; likewise, it increased from 0 to 17.0 MPa and then decreased to 4.8 MPa for FA1CCR1-L-50 °C.

### 3.2. Microstructure of FA/CCR-Based Geopolymer

#### 3.2.1. XRD Results

**Figure 3:**
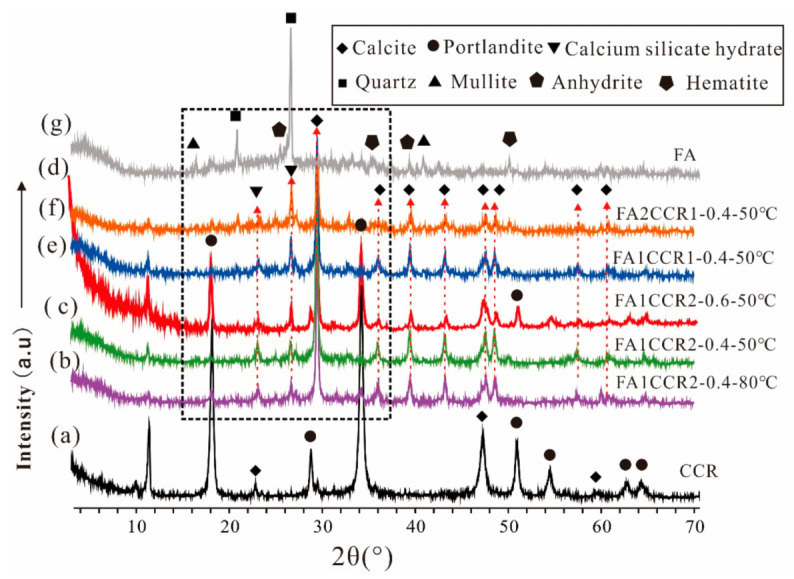
The XRD patterns of raw materials and FA/CCR-based geopolymers. (**a**) CCR; (**b**) FA1CCR2-0.4-80 °C; (**c**) FA1CCR2-0.4-50 °C; (**d**) FA1CCR2-0.6-50 °C; (**e**) FA1CCR1-0.4-50 °C; (**f**) FA2CCR1-0.4-50 °C; (**g**) FA.

The XRD patterns of CCR and FA are plotted in Figure 3a,g, respectively. As can be seen, a broad reflection spanning 16° (2θ) to 38° (2θ) was revealed in the XRD pattern of FA (Figure 3g), demonstrating the presence of an amorphous phase. The amorphous phase in FA possessed pozzolanic reactivity [46]. Additionally, the FA also contained many crystalline phases, these including quartz, anhydrite, mullite, and hematite. These phases are stable, which shows less reactivity. In Figure 3a, we see that the CCR was mainly composed of portlandite, with only little amounts of calcite. Therefore, the Ca present in CCR was mainly in the form of Ca(OH)_2_, which suggested CCR could provide sufficient alkalinity and calcium during the geopolymerization process.

**Figure 4:**
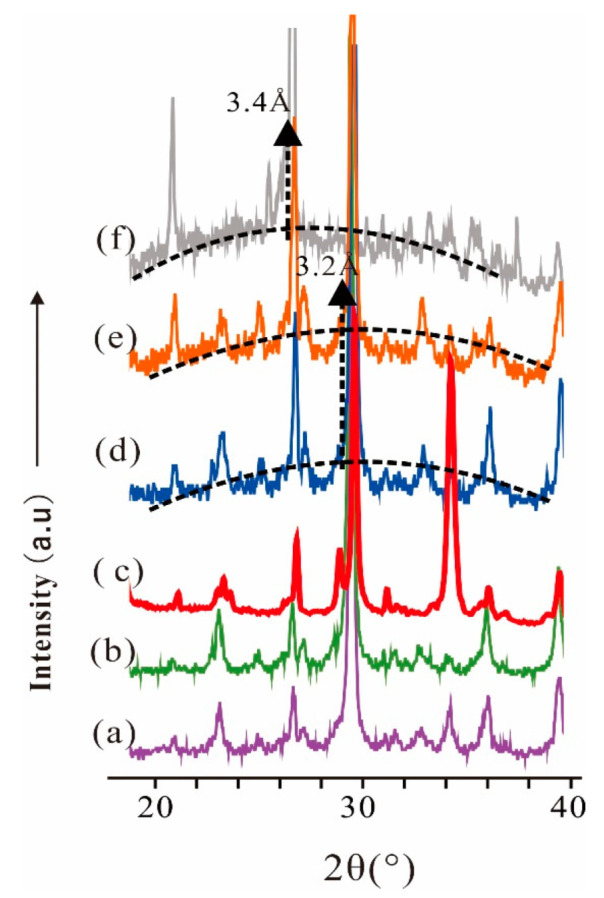
The XRD patterns of FA and FA/CCR-based geopolymers enlarged at 20° (2θ) to 40° (2θ). (**a**) FA1CCR2-0.4-80 °C; (**b**) FA1CCR2-0.4-50 °C; (**c**) FA1CCR2-0.6-50 °C; (**d**) FA1CCR1-0.4-50 °C; (**e**) FA2CCR1-0.4-50 °C; (**f**) FA.

In Figure 3b–f are displayed the XRD patterns of FA/CCR-based geopolymers with different preparation conditions; Figure 4 shows a magnified view of the rectangular region drawn in the Figure 3 graphic. For FA2CCR1-0.4-50 °C (Figure 4e) and FA1CCR1-0.4-50 °C (Figure 4d), both featured one pronounced broad reflection centered at 29° (2θ), in which the angle regions of the reflection center exceeded those of FA (Figure 4f). This broad reflection suggests the existence of an amorphous phase, that may correspond to the coexistence of calcium alumina silicate hydrate (C-A-S-H) and calcium silicate hydrate (C-S-H) [47,48]. This result also demonstrated that FA did indeed react with CCR to form a geopolymer network during the mixing with water [49,50]. In stark contrast, there was no such pronounced broad reflection in the XRD patterns of FA1CCR2-0.6-50 °C (Figure 4c), FA1CCR2-0.4-50 °C (Figure 4b), and FA1CCR2-0.4-80 °C (Figure 4a), a finding perhaps related to the presence of many unreacted minerals. Besides, the low content of FA for raw materials also was responsible for the low content of geopolymer, resulting in the inconspicuous broad reflection.

Except for that broad reflection, no other obvious diffraction difference was discernible among all the FA/CCR-based geopolymers tested. Due to its high chemical stability, the quartz still remained intact after the reaction (Figure 3b–f). Meanwhile, Ca(OH)_2_ were consumed with the decreased content of Ca(OH)_2_ in all specimens. As the FA/CCR ratio decreased, the content of unreacted Ca(OH)_2_ increased. Notably, the intensity of Ca(OH)_2_ in the XRD pattern of FA1CCR2-0.6-50 °C was greater than those of the other specimens. This result demonstrated the geopolymer with a water/binder ratio of 0.6 also contained more Ca(OH)_2_ than did the other geopolymers, suggesting excessive water may reduce the alkalinity and hinder the geopolymerization process. Furthermore, new crystalline calcite formed after the reaction, this arising from the geopolymer product reacting with atmospheric CO_2_. Importantly, the curing temperature and water/binder ratio used did not significantly change the mineralogical composition of any of the geopolymer specimens.

#### 3.2.2. FTIR Results

**Figure 5:**
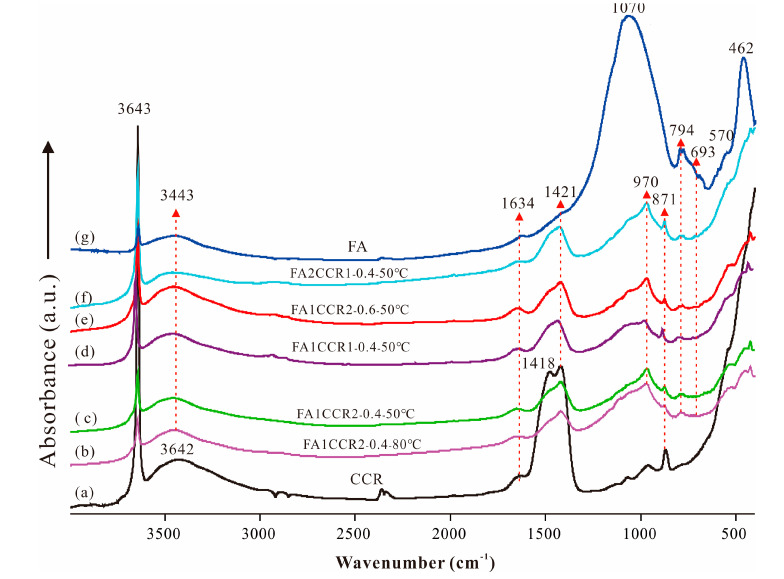
The FTIR spectra of raw materials and FA/CCR-based geopolymers. (**a**) CCR; (**b**) FA1CCR2-0.4-80 °C; (**c**) FA1CCR2-0.4-50 °C; (**d**) FA1CCR1-0.4-50 °C; (**e**) FA1CCR2-0.6-50 °C; (**f**) FA2CCR1-0.4-50 °C; (**g**) FA.

Figure 5 plots the FTIR spectra of FA and CCR, and the FA/CCR-based geopolymers. In Figure 5g, the sharp peak at 3642 cm^−1^ was ascribed to the stretching vibration of O-H from Si-OH, while the broad peaks at 3443 cm^−1^ and 1634 cm^−1^ were respectively attributable to O-H stretching and the bending vibrations of physically adsorbed water, respectively [51]. The broad peak located at 1070 cm^−1^ was associated with Si-O-T (T: Si or Al) and its symmetric stretching vibration, whereas the peak at 461 cm^−1^ likely corresponds to the bending vibration of an in-plane Si-O [46,52]. Two other peaks, at 794 cm^−1^ and 693 cm^−1^, were assigned to the Si-O-Si vibration of quartz, while that at 570 cm^−1^ probably arose from the Si-O-Al symmetric stretching vibration of mullite [53]. In the FTIR spectrum of CCR (Figure 5a), there also existed sharp peak at 3642 cm^−1^, but it should correspond to the O-H stretching vibration of Ca-OH. The C-O asymmetric stretching vibration of CaCO_3_ occurred at 1418 cm^−1^, while the CO_3_^2−^ deformation vibration occurred in 871 cm^−1^ [46,54]. Taken together, these results confirm the presence of Ca(OH)_2_ and CaCO_3_ in the CCR.

In Figure 5b–f the FTIR spectra of the tested FA/CCR-based geopolymers appear. The main band for FA, with a wavenumber of 1070 cm^−1^, shifted to a lower wavenumber of approximately 970 cm^−1^ after the reaction, which confirmed the geopolymer formation, i.e., the partial dissolution of FA reorganized to form the C-(A)-S-H network [55]. Yet there were no pronounced spectrum differences among all the FA/CCR-based geopolymers, whose peaks could all be found in the FTIR spectra of FA and CCR. This similarity may have two explanations. (1) The inadequate complete dissolution of FA; for example, the sharp peak at 3643 cm^−1^ indicated the alkalinity of CCR was too low to dissolve FA and so the Si-O was left intact after reacting with CCR. (2) Some typical vibrations of Si–O–T (i.e., at 693 cm^−1^) from the geopolymer were overlapped by those from unreacted minerals; indeed, it is hard to discern the vibration of Si–O–T between unreacted minerals and FA/CCR-based geopolymers [23].

#### 3.2.3. TGA-DTG Results

**Figure 6:**
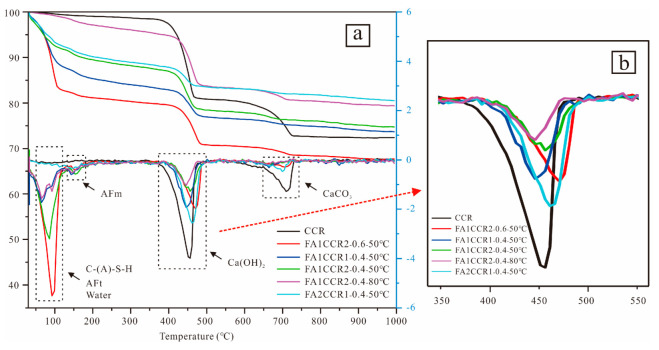
(**a**) The TGA and DTG curves of CCR and the FA/CCR-based geopolymers. (**b**) the enlarged scale between 350–550 °C of DTG curve.

Figure 6 displays the TGA-DTG curves of the raw material and FA/CCR-based geopolymers, showing the weight loss of the specimens in response to heating. In the TGA curve of CCR, two obvious weight loss events were found, these occurring at 350 °C to 500 °C and 650 °C to 750 °C, presumably caused by the decomposition of Ca(OH)_2_ (Figure 6b) and CaCO_3_, respectively [54]. Notably, from the DTG curves, it is evident the intensity of the peak corresponding to the decomposition of Ca(OH)_2_ was reduced significantly after the reaction, indicating that Ca(OH)_2_ had been consumed while reacting with FA. Significant weight losses of FA/CCR-based geopolymers were also found between room temperature to approximately 200 °C, which likely corresponds to the decomposition of hydration products, namely C-(A)-S-H, ettringite (AFt), and monosulfate (AFm) that formed in the reaction [56,57] and lost free water [58]. Among them, the peak around 110 °C is attributable to an overlapping of the released interlayer water in C-S-H and decomposition of C-S-H and AFt [59], while the existence of a small peak around 150 °C suggests the formation of AFm [60].

The FA1CCR2-0.6-50 °C geopolymer underwent the greatest weight loss at 100 °C, this resulting from the inclusion of high amounts of free water. Furthermore, the intensity of this peak increased as the FA/CCR ratio decreased, thus demonstrating that more hydration products were formed for same water content. As reported previously, C-S-H and AFt are deemed mainly responsible for the strength development [57,61]; hence, this could explain the high compressive strength of FA1CCR2-0.4-50 °C. When the curing temperature increased to 80 °C, the intensity of this peak declined significantly because high amounts of water were evaporated during the curing process.

### 3.3. Morphology and Chemical Composition of FA/CCR-Based Geopolymers

**Figure 7:**
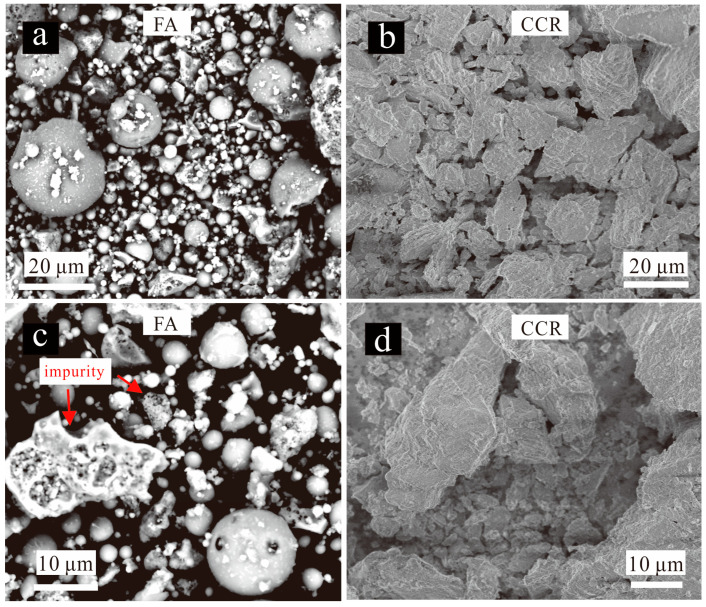
SEM images of FA and CCR. (**a**,**c**) CCR; (**b**,**d**) FA.

Figure 7a,b presents the SEM images of FA and CCR. The FA mainly consisted of small spherical particles carrying in size (Figure 7a,c), whose radius ranged from 2.5 μm to 25.8 μm. The FA particles included cenospheres, pleropheres, as well as some impurities. By contrast, the CCR consisted mostly of many large particles irregular in shape (Figure 7b,d).

**Figure 8:**
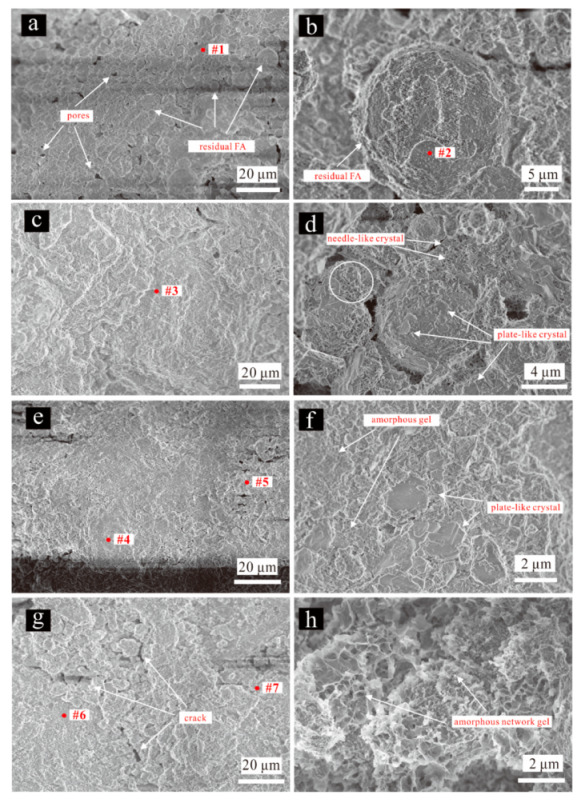
The SEM images of FA/CCR-based geopolymers. (**a**,**b**) FA2CCR1-0.4-50 °C; (**c**,**d**) FA1CCR1-0.4-50 °C; (**e**,**f**) FA1CCR2-0.4-50 °C; (**g**,**h**) FA1CCR2-0.4-80 °C.

In Figure 8, the panel pairs 8a-b, 8c-d, 8e-f, and 8g-h show the microscopic morphology of FA/CCR-based geopolymers of FA2CCR1-0.4-50 °C, FA1CCR1-0.4-50 °C, FA1CCR2-0.4-50 °C, and FA1CCR2-0.4-80 °C respectively at low and high magnification. One can see from Figure 8a that many FA particles were loosely cemented together, which indicated the content of crystalline hydration products and amorphous gel was low. At higher magnification, the surface of FA particles was only slightly corroded (Figure 8b), confirming that CCR harbors insufficient alkalinity to dissolve FA for FA2CCR1-0.4-50 °C.

When the FA/CCR ratio was lowered to 1:1 and 1:2, the matrix of specimens got compacted and became denser, mainly because more of the geopolymer had formed (Figure 8c,e), this resulting from the greater alkalinity introduced by CCR. Therefore, the compressive strength increased. At high magnification, there were many plate-like and needle-like crystals found distributed in the surface of FA and the geopolymer matrix, not unlike those observed in previous studies e.g., [2,54,62,63]. According to Wang et al. [64], the plate-like crystals with a hexagonal shape should be Friedel’s salt crystals. Needle-like crystals present in hydrated cement are often attributed to ettringite [63,65]; however, in our study, due to the low content of sulfur (Table 1), it seems the content of ettringite formed during geopolymerization was rather low. Instead, following Nonat [66] and Bo et al. [67], this type of needle-like crystal might correspond to C-S-H gels or CaCO_3_.

When the curing temperature was increased to 80 °C, microcracks and voids ensued and the geopolymer matrix became loosely bound, thus weakening its compressive strength. This is because too high a curing temperature will not only cause the rapid hardening of geopolymeric paste without sufficient dissolution occurring, but also form more pores as more evaporation occurs [19]. At high magnification, flocculating species were observed in the matrix, this being consistent with the morphology of C-S-H gel [67,68].

**Figure 9:**
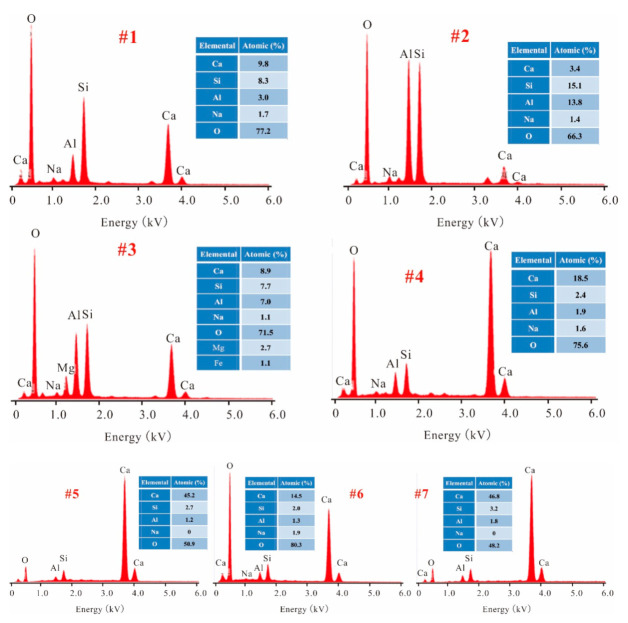
The EDX spectra of selected spots #1–7 indicated by circles in Figure 8.

The EDX analysis was performed on selected spots #1–7, these indicated in Figure 8, for which the energy spectra detection results are displayed in Figure 9. We found that the main elements of spot 1 were Al, Ca, Si, and O, whose mass ratio exceeded 98%. This result is further evidence that C-A-S-H gel was formed through the reaction between FA and CCR. Differently, however, is spot 2 which featured the main elements of Si, Al, and O, this confirming that the detected spherical particles were unreacted FA. The EDX spectra of spots 3 and 4 indicated that the matrix of FA1CS1-0.4-50 °C and FA1CS2-0.4-50 °C each contained the C-A-S-H gel. When compared with spot 1, it becomes evident that with an increased content of CCR, the Ca/Si and Ca/Al molar ratio respectively increased from 0.83 to 5.40 and from 2.27 to 6.58. Crucially, FA1CS2-0.4-80 °C had a chemical composition of C-A-S-H similar to that of FA1CS2-0.4-50 °C; hence, increasing the curing temperature will not significantly change the composition of C-A-S-H gel. In addition, worth noting is that in some areas of FA1CS2-0.4-50 °C and FA1CS2-0.4-80 °C (spots 5 and 7), the Ca content was very high, reaching approximately 50%. Due to excess CCR, a Ca-rich matrix ensues, containing little amounts of Si and Al elements. Therefore, except for the C-(A)-S-H gel, these areas may also harbor other Ca-containing species, such as CaCO_3_, Ca(OH)_2_ and C-S-H.

Collectively, the above results clearly demonstrate that it is feasible to use CCR and FA as raw materials to prepare a geopolymer without having to supplement it with an extra alkaline solution. When the FA/CCR ratio is 1:2, the 28-d compressive strength reached approximately 17.5 MPa, which can be used for some construction materials [69]. The CCR provided alkali that can effectively react with FA to form the C-(A)-S-H gels. Moreover, the Ca(OH)_2_ in the excess CCR are able to react with atmospheric CO_2_ to form CaCO_3_, which also enhances the strength of the products. Therefore, using CCR in the preparation of FA-based geopolymers can recycle CCR, mitigate adverse environmental impacts, and foster the development of high value-added products.

### 3.4. Summary and Final Discussion

The preceding results show that the microstructure and compressive strength of FA/CCR-based geopolymers were greatly influenced by FA/CCR ratio, curing temperatures, and water/binder ratio. When the FA/CCR ratio was set as 1:2, water/binder ratio was set as 0.4, followed by cured at 50 °C, the maximum 28 d compressive strength of as-obtained geopolymer can reach higher than 17.5 MPa. However, compared to normally alkali-activated FA/ground granulated blast furnace slag (GGBFS)-based geopolymer, this compressive strength seems much lower. For example, Chen et al. [70] optimized the alkali-activated FA/slag-based geopolymer by response surface method. The 3d and 28 d compressive strengths were 28.10 MPa and 54.69 MPa, respectively. Dehghani et al. [71] investigated the effect of initial SiO_2_/Al_2_O_3_ ratio on the FA/GGBFS-based geopolymer and reported that the compressive strength firstly increased and then decreased as the SiO_2_/Al_2_O_3_ ratio increased, in which the maximum 91 d compressive strength reached 45.3 MPa when SiO_2_/Al_2_O_3_ ratio was 3.37. According to Sasui et al. [72], the compressive strength of FA/GGBFS-based geopolymer could reach approximately 60 MPa using NaOH and Na_2_SiO_3_ as alkali activator.

Although the compressive strength of less than 20 MPa for FA/CCR-based geopolymer may limit its application, it can be also applied as construction materials in many fields. For instance, the compressive strength of pavement base in China can be around 5 MPa [69], which is easy to achieve using geopolymer derived from FA/CCR. Zhao et al. [73] studied the FA/soda residue-based geopolymer for goaf backfill, in which the highest compressive strength was only 4.2 MPa. What’s more, the minimum compressive strength of brick is required as 10 MPa, whereas those higher than 15 MPa of brick can be used as load bearing wall. That is, FA/CCR-based geopolymers can be used for preparing bricks. Moreover, the mechanical properties can be further improved by pretreating the raw materials such as calcination or grinding. More importantly, the when using CCR to replace GGBFS, the alkali activator can be replaced by water for preparing geopolymer, which became much more environmental friendly and economic.

In this study, we found that although the XRD and FTIR results of FA/CCR-based geopolymers prepared by different conditions did not change noticeably, their compressive strengths and microstructure changed obviously. For FA/CCR ratio, it can be found that the content of CCR should be high enough to provide sufficient alkali to go through the geopolymerization of FA. After addition of water, FA can react with CCR to form geopolymer with C-A-S-H gels (Figure 9). However, as the increased FA content, the unreacted FA increased due to the incomplete reaction between FA and CCR, as can be seen in the SEM images of specimens (Figure 8). For curing temperature, it can be seen that increasing the temperature significantly increased the early compressive strength but detrimental to the late compressive strength if the curing temperature was too high. However, the chemical composition appeared to remain unchanged (Figure 9). Geopolymer cured at 60 °C appeared to exhibit suitable early and late compressive strengths. However, the geopolyemrs cured at 80 °C possessed less water content (Figure 6) and loosely-bond microstructure (Figure 8), which may respond for its low compressive strength. For water/binder ratio, the water content should not be too low to provide sufficient workability. On other hand, increasing the water content resulted in the decrease of early and late compressive strength, mainly because increasing water diluted the alkalinity of the whole system as well as created many pores resulting from evaporation during curing. The high content of water (Figure 6) may improve the shrinkage properties of FA/CCR-based geopolymers, but the low compressive strength indicated it’s not suitable to increase the water/binder ratio for preparing geopolymer.

## 4. Conclusions

Fly ash contains reactive SiO_2_ and Al_2_O_3_ and CCR is a solid waste by-product that possesses high alkalinity. In this study, FA incorporated with CCR was used as raw material to prepare geopolymeric materials with a dense microstructure and desirable mechanical properties. Although the FA/CCR ratio was as low as 1:2, the 28 d compressive strength of a FA/CCR-based geopolymer could reach 17.5 MPa, favoring the recycling of CCR. The formation of a high amount of C-(A)-S-H was conducive to the microstructure’s densification and improvement of its compressive strength. What is more, traces of AFt and AFm were also found in the geopolymer products. Nonetheless, curing at too high a temperature (80 °C) or using too much water (water/binder ratio ≥ 0.6) during geopolymerization generated a loose microstructure and reduced compressive strength.

Our results demonstrate that FA incorporated with CCR is a promising candidate for preparing geopolymers capable of suitable performance without the addition of any extra alkali. This offers great potential for reducing the environmental impact of both FA and CCR and for developing high-value-added products for the associated industries.

## Figures and Tables

**Table 1:** The chemical composition of CCR and FA. Values are relative proportions (wt%).

	SiO_2_	Al_2_O_3_	Fe_2_O_3_	K_2_O	MgO	CaO	Na_2_O	TiO_2_	SO_3_	Others
**FA**	53.63	21.71	7.96	1.42	1.17	10.80	1.21	0.86	-	1.24
**CCR**	2.43	0.69	0.35	-	0.11	94.81	0.47	-	0.56	0.58

**Table 2:** The mix proportions used to prepare the FA/CCR-based geopolymers.

Specimen	FA	CCR	Water	Water/Binder Ratio	Curing Temperature
FA1CCR2-0.4-50 °C	80 g	160 g	96 g	0.4	50 °C
FA2CCR3-0.4-50 °C	96 g	144 g	96 g	0.4	50 °C
FA1CCR1-0.4-50 °C	120 g	120 g	96 g	0.4	50 °C
FA3CCR2-0.4-50 °C	144 g	96 g	96 g	0.4	50 °C
FA2CCR1-0.4-50 °C	160 g	80 g	96 g	0.4	50 °C
FA1CCR2-0.4-40 °C	80 g	160 g	96 g	0.4	40 °C
FA1CCR2-0.4-60 °C	80 g	160 g	96 g	0.4	60 °C
FA1CCR2-0.4-80 °C	80 g	160 g	96 g	0.4	80 °C
FA1CCR2-0.3-50 °C	80 g	160 g	72 g	0.3	50 °C
FA1CCR2-0.5-50 °C	80 g	160 g	120 g	0.5	50 °C
FA1CCR2-0.6-50 °C	80 g	160 g	144 g	0.6	50 °C
FA1CCR1-0.3-50 °C	120 g	120 g	72 g	0.3	50 °C
FA1CCR1-0.5-50 °C	120 g	120 g	120 g	0.5	50 °C
FA1CCR1-0.6-50 °C	120 g	120 g	144 g	0.6	50 °C
